# Evaluation of Acrylamide in Food from China by a LC/MS/MS Method

**DOI:** 10.3390/ijerph9114150

**Published:** 2012-11-14

**Authors:** Yong-Hong Chen, En-Qin Xia, Xiang-Rong Xu, Wen-Hua Ling, Sha Li, Shan Wu, Gui-Fang Deng, Zhi-Fei Zou, Jing Zhou, Hua-Bin Li

**Affiliations:** 1 Guangdong Inspection and Quarantine Technology Center, Guangzhou 510623, China; Email: cathy66@21cn.com (Y.-H.C.); zfzhou@163.com (Z.-F.Z.); 2 School of Public Health, Guangdong Medical College, Dongguan 510234, China; Email: enqinxia@yahoo.com; 3 Key Laboratory of Marine Bio-Resources Sustainable Utilization, South China Sea Institute of Oceanology, Chinese Academy of Sciences, Guangzhou 510301, China; 4 Guangdong Provincial Key Laboratory of Food, Nutrition and Health, Department of Nutrition, School of Public Health, Sun Yat-Sen University, Guangzhou 510080, China; Email: lingwh@mail.sysu.edu.cn (W.-H.L.); 811858432@qq.com (S.L.); wushansw@sina.com (S.W.); misyfly@163.com (G.-F.D.); jingzhou@163.com (J.Z.)

**Keywords:** acrylamide, LC/MS/MS, food analysis, food safety, public health

## Abstract

Acrylamide is potential carcinogenic compound that possesses neurotoxicity activity. In this study, the levels of acrylamide in 123 selected food samples from China was evaluated using a LC/MS/MS method. One hundred and fifteen (115) out of 123 samples showed positive levels of acrylamide in the range of 0.41 to 4,126.26 µg/kg. Generally, the highest acrylamide levels were found in fried products, such as potato, prawn strips and rice crust, with average values of 604.27, 341.40, and 201.51 µg/kg, respectively. Heated protein-rich food also showed some acrylamide content (ranging from 2.31 to 78.57 µg/kg). The results revealed that a potential acrylamide public health risk occurred in processed snacks, as well as the food consumed daily. This study supplied new information on acrylamide content of a variety of heat-treated foods from China.

## 1. Introduction

**Figure 1:**
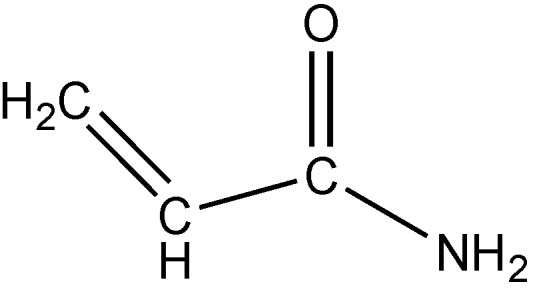
The chemical ostructure of acrylamide.

Many hazardous compounds have been identified in food, and they usually come from a polluted environment, such as polycyclic aromatic hydrocarbons (PAHs) and heavy metals [1,2]. Recently, some contaminants in food, such as PAHs, chloropropanols and acrylamide, which are generated in food during heat treatment procedures, have attracted the public’s concerns all over the World [3,4,5]. Acrylamide (CH_2_=CH-CONH_2_) is a chemical compound used to synthesize polyacrylamide. The chemical structure was illustrated in Figure 1. In animal assays, acrylamide induced significant characteristic neurotoxic symptoms in rats, such as decreases in grip strength and locomotor activity [6,7]. Acrylamide can increase the risk of tumors of the mammary glands, central nervous system, thyroid gland-follicular epithelium, uterus, colon and clitoral gland in rats [8,9]. Acrylamide also has other negetive effects on public health, such as decreasing immune and blood systems [10,11,12]. Its potential health risks may extend to human beings, and it has been considered by the International Agency for Research on Cancer as ‘‘probably carcinogenic to humans’’ (group 2A) [11,13]. Since 2002, high levels of acrylamide have been found in some foods, such as fried, baked, grilled or toasted foods [14,15]. The high acrylamide level in some foods has attracted public concern as a thermal process-induced contaminant. The potential risk to public health of acrylamide in food has been considered by many government agencies and national authorities.

Extensive surveillance on acrylamide levels in foods has been conducted and reported from several countries [14,16,17,18,19,20], but no such report on systematic surveillance of acrylamide levels in food from China could be found in the World scientific literature, except for several papers reporting new analytical methods and applying them to the determination of acrylamide in some foods to verify the applicability of the method [21,22]. 

Guangzhou City, with its rapid economic growth and high development, is an important industrial, cultural and communication center of China. There are many people living in Guangzhou, and they come from Hong Kong, Macau, Taiwan and different provinces of China Mainland as well as many countries around the World. The food sold in Guangzhou also comes from different places in China, and could be considered as a representative of typical Chinese food. The quality of food sold in Guangzhou not only relates to the health of Chinese people, but also correlates with the health of visiting guests from other countries. Moreover, in Guangzhou, fast food or snack, such as fried food in KFC and roasted food in bakery, are widely consumed by the public, especially by young people at work due to the quick pace of life, as well as children and teens due to habit. Thus, it is very important to evaluate the acrylamide levels in foods from Guangzhou. Thus, this study has been carried out to examine the level of acrylamide in 123 selected foods from Guangzhou to supply new information on acrylamide levels in a variety of foods from China.

## 2. Experimental

### 2.1. Chemicals

Standard acrylamide was obtained from Sigma Aldrich (Diesenhofen, Germany). Methanol, formic acid, *n*-hexane, acetone, ethyl acetate and acetonitrile were of HPLC grade and obtained from Merck (Darmstadt, Germany). Sodium chloride, ammonium sulfate and potassium ferrocyanide were of analytical grade, and purchased from Tianjin Chemical Factory (Tianjin, China). Ultrapure water (18.2 mΩ·cm) obtained from a Millipore purification system (Billerica, MA, USA) was used throughout the experiment. 

### 2.2. Apparatus

The microwave-assisted extraction was carried out in an X-100A microwave extraction device (Xianghu Instrumental Company, Beijing, China) with a microwave power of 1,000 W and a temperature monitor as well as microprocessor programmer software to control the performance parameters of the device, *i.e.*, microwave power, temperature and running time. 

### 2.3. Sample Treatment

A total 123 food samples were collected from different supermarkets and restaurants in Guangzhou, China. The collected samples were transported to the laboratory and stored at −20 °C. The samples (about 2 g) were accurately weighed into a 50 mL centrifuge tube, and then mixed with an appropriate amount of ultra-pure water (20 mL). For food products with high fat content (crisps, chips, roasted nuts, olive, hazelnut paste, peanut paste, traditional desserts, meals), a defatting step should be performed by using *n*-hexane extraction (20 mL, twice) at room temperature before adding the water. Two grams of ammonium sulfate and 1.1 g potassium ferrocyanide were added. The mixture was vortexed for 2 min, then irritated by microwave for 10 min at 400 W power. After centrifugation at 7,000 g for 10 min at 4 °C, the supernatant was collected. Then, 6.5 g of sodium chloride and 20 mL of ethyl acetate were added, and the mixture was vortexed for 2 min. After centrifugation at 3,500 g for 10 min at 4 °C, the upper ethyl acetate layer was collected. Then, the ethyl acetate extract was dried by nitrogen gas, and the residue was redissolved in 0.8 mL of ultrapure water in a 2.5 mL capped centrifuge tube. Twenty µL of the aqueous sample was injected into the LC-MS-MS system for the analysis.

### 2.4. Acrylamide Analysis

LC-MS-MS analysis of acrylamide was performed with an Agilent 1100 high-performance liquid chromatography (HPLC) system (Santa Clara, CA, USA) consisting of a quaternary pump, an autosampler and a temperature-controlled column oven, coupled to an Agilent 1100 MS detector equipped with an electrospray ionization interface. The analytical column was a Kinetex C18 column (100 × 4.6 mm, 2.6 µm particle size) from Phenomenex (Torrance, CA, USA). The mobile phase was acetonitrile and 1/1,000 formic acid aqueous solution (30:70, v/v) with a flow-rate of 0.2 mL/min. The temperature of column was kept at 25 °C. Data acquisition was performed, with a delay time of 8 min, in a selected ion-monitoring (MRM) mode using the following interface parameters: a drying gas (N_2_, 665 Pa) flow of 11 L/min, nebulizer pressure of 300 Pa, drying gas temperatures of 350 °C, a capillary voltage of 11 kV and a fragmenter voltage of 40 eV. 

### 2.5. Statistical Analysis

The statistical analyses were carried out with SPSS Window version 10.0 (SPSS, Chicago, IL, USA). Data for acrylamide levels of analysis of variance was performed to test the difference in acrylamide levels depending on food type. 

## 3. Results and Discussion

### 3.1. Validation of the Analytical Method

Prior to analysis of acrylamide, the LC-MS-MS method was validated to ensure the quality of the data. The linearity of the calibration curve was checked by a series of standard solutions of acrylamide ranged from 20 to 200 µg/L at seven different concentrations. The recoveries and the relative standard deviation were studied by the prepared samples spiked with different concentrations of acrylamide (0.8, 1.0 and 1.5 µg/kg). The results are expressed in Table 1. The method showed a good linearity in concentrations ranging from 20 to 200 μg/mL with a correlation coefficient of 0.9993. The limit of detection was 2.4 μg/mL based on a signal/noise ratio of 3:1 [23]. The recoveries ranged from 91% to 97%, and the relative standard deviation was 3.5%. 

**Table 1:** The linear range, limit of detection, recovery and precision of the method.

Linear range	R^2^	LOD (μg/kg)	RSD (%)	Recovery (%)
20~200 μg/L	0.9988	2.4	3.5	91~97

The contents of acrylamide in several food samples were simultaneously determined by LC/MS/MS in this study and gas chromatography (GC) based on the National Standards of Peoples Republic of China GB/T 5009.204-2005. No significant difference between the results of two methods was observed (*p* < 0.05). 

### 3.2. The Occurrence and Levels of Acrylamide in Foods

**Figure 2:**
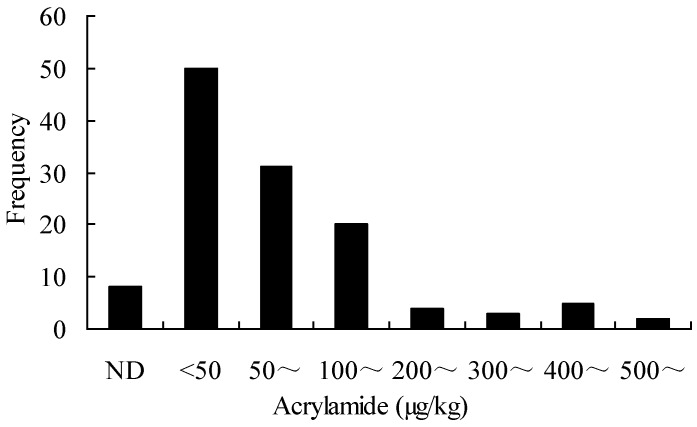
Frequency distribution of acrylamide content in food.

A total 123 samples classified into 15 categories were analyzed for amounts of acrylamide. The occurrence and levels of acrylamide in the samples are shown in Table 2, and the frequency distribution of acrylamide content in foods is illustrated in Figure 2. In total 115 out of 123 samples showed positive levels of acrylamide ranging from 0.41 to 4,126.26 µg/kg, and the levels of acrylamide in 34 samples were more than 100 µg/kg. That is, acrylamide was present in 93.5% of the total samples analyzed, and 27.6% of the total samples contained over 100 µg/kg of acrylamide. 

**Table 2:** The acrylamide contents in different kinds of food.

Category	Samples Tested	Samples Positive	Level of Acrylamide (µg/kg)			
			Mean	SD	Min	Max
Soybean paste	4	4	13.70	8.39	4.08	24.40
Processed meat	6	6	22.62	20.10	2.31	49.06
Rice roll and noodle	11	9	23.22	15.19	9.12	52.09
Cooked meat	5	5	25.03	21.97	2.71	78.57
Sauted nut	8	7	25.14	19.09	4.04	54.66
Roasted bread	5	5	36.72	26.54	10.50	67.19
Wafer biscuit	6	5	44.92	33.86	10.36	86.76
Roasted rice cake	6	6	68.34	65.75	16.82	196.46
Pancake	8	7	70.33	127.86	1.88	352.90
Roasted biscuit	24	23	97.57	103.71	0.41	484.17
Crisp	11	9	137.91	119.68	17.39	398.23
Fried flour snack	8	8	131.73	122.75	39.12	432.92
Fried rice crust	8	8	201.51	122.62	100.46	491.76
Fried prawn strips	4	4	341.40	122.95	166.25	439.44
Fried potato	9	9	604.27	1,327.87	58.40	4,126.26
Total	123	115	94.16	54.74	0.41	4,126.26

There was a great variation between different food types. Four samples of soybean paste were found positive, and their acrylamide contents (4.08–24.40 µg/kg) were the lowest among the samples tested. The samples of processed and cooked meat, rice roll, noodle, roasted bread, wafer biscuit, and sauteed nuts contained high levels of acrylamide, with average values of 22.62–44.92 µg/kg for the 37 positive samples out of the 41 samples analyzed. Roasted cake and biscuits showed markedly high levels of acrylamide, with average values of 68.34–97.57 µg/kg in 36 positive samples out of 38 samples analyzed. In this study, the highest levels of acrylamide contents, with an average of 131.73–604.27 µg/kg, were found in 38 positive samples of processed and cooked crisps, fried flour, rice, prawns, and potato products, and only 2 of the 40 samples were below the LOD. Similar results were seen in the previous research, which might be accounted for by the differences in cooking and processing parameters, methods, type and quality of raw materials, as well as formulations [24]. The effect of conditions of cooking and processing on the occurrence of the contaminant was considerable, and research in the relationship between levels of acrylamide and those parameters would shed some light on the options for reduction of the acrylamide content in foods. 

In the present study, the occurrence of acrylamide in protein-rich foods was also investigated. The results showed that all heated meat foods were found positive, with the largest value of 78.57 µg/kg. This indicated that the occurrence of the acrylamide contamination occurred in the daily cooking procedures, but the heated protein-rich foods usually contained lower levels of acrylamide than carbohydrate-rich foods. The previous reports showed that acrylamide mainly originates from the Maillard reaction of the amino acid asparagine with reducing sugars [15,25]. Some studies have demonstrated that sugars could play an important role in the formation of acrylamide in some foods, and glucose might be the most effective [26,27]. 

The distribution and contents of acrylamide in food in some previous studies are shown in Table 3. In the present study, the levels of acrylamide in samples were equal or comparable to the similar samples in the previous research. As reported in other countries and regions, acrylamide also occurred in the daily heated foods in Guangzhou City, such as heated protein-rich foods, rice cakes, instant noodles, and biscuits (Table 2), which made up a large part of the daily public food consumption and therefore showed some risk to public health. The highest contents of acrylamide are often present in other carbohydrate-rich foods heated at higher temperatured, such as the roasted and fried snacks, which show different consumption patterns for people of various ages. 

**Table 3:** The distribution and contents of acrylamide in foods.

Sample	Acylamide Level	References
Breakfast cereals	<62–803 μg/kg	[18]
Carbohydrate-rich food	<20–2,528 μg/kg	[16]
Potato crisps	325 μg/kg	[17]
Espresso coffee	11.4–36.2 μg/L	[20]
Chocolate	102 μg/kg	[19]
Pizza, minced meat, fried bacon	0–1,480 μg/kg	[28]
Baby strained prunes	75–265 μg/kg	[29]

Those kinds of foods have a heavy contribution to acrylamide intake due to the fact they are usually a part of the normal diet, breakfast or supplemental food for children and teens, as well as for persons at work. As a result, it is urgent to employ abatement strategies for acrylamide in food for public health. 

## 4. Conclusions

In a total of 123 samples, acrylamide was detected in 115 samples at levels of 0.41 to 4,126.26 µg/kg. The highest acrylamide levels were found in fried products, such as potato, prawn strips and rice crust, which indicated the general risk of consumer exposure to acrylamide from foods. The results revealed that a widespread potential risk from acrylamide for public health occurred in processing snacks, as well as other foods treated with lower temperatures than fried processing. This investigation supplied important information on acrylamide in food for public health experts, food policy makers and consumers.
